# PRMT5 Is Required for Bovine Leukemia Virus Infection In Vivo and Regulates BLV Gene Expression, Syncytium Formation, and Glycosylation In Vitro

**DOI:** 10.3390/v12060650

**Published:** 2020-06-16

**Authors:** Wlaa Assi, Tomoya Hirose, Satoshi Wada, Ryosuke Matsuura, Shin-nosuke Takeshima, Yoko Aida

**Affiliations:** 1 Laboratory of Viral Infectious Diseases, Department of Computational Biology and Medical Sciences, Graduate School of Frontier Sciences, The University of Tokyo, 2-1 Hirosawa, Wako, Saitama 351-0198, Japan; wlaa.pharma92@yahoo.com (W.A.); kamaboko517tmp@gmail.com (T.H.); oinari.atsuage@gmail.com (R.M.); takesima@jumonji-u.ac.jp (S.-n.T.); 2 Viral Infectious Diseases Unit, RIKEN, 2-1 Hirosawa, Wako, Saitama 351-0198, Japan; 3 Photonics Control Technology Team, RIKEN Center for Advanced Photonics, 2-1 Hirosawa, Wako, Saitama 351-0198, Japan; swada@riken.jp; 4 Department of Food and Nutrition, Jumonji University, Niiza, Saitama 352-8510, Japan; 5 Nakamura Laboratory, Baton Zone program, Riken Cluster for Science, Technology and Innovation Hub, 2-1 Hirosawa, Wako, Saitama 351-0198, Japan

**Keywords:** bovine leukemia virus, PRMT5, proviral load, BLV gene expression, PRMT5 inhibitor, gp51, glycosylation, syncytia formation

## Abstract

Bovine leukemia virus (BLV) is the causative agent of enzootic bovine leukosis, which is the most common neoplastic disease of cattle and is closely related to human T-cell leukemia viruses. We investigated the role of a new host protein, PRMT5, in BLV infection. We found that PRMT5 is overexpressed only in BLV-infected cattle with a high proviral load, but not in those with a low proviral load. Furthermore, this upregulation continued to the lymphoma stage. PRMT5 expression was upregulated in response to experimental BLV infection; moreover, PRMT5 upregulation began in an early stage of BLV infection rather than after a long period of proviral latency. Second, siRNA-mediated PRMT5 knockdown enhanced BLV gene expression at the transcript and protein levels. Additionally, a selective small-molecule inhibitor of PRMT5 (CMP5) enhanced BLV gene expression. Interestingly, CMP5 treatment, but not siRNA knockdown, altered the gp51 glycosylation pattern and increased the molecular weight of gp51, thereby decreasing BLV-induced syncytium formation. This was supported by the observation that CMP5 treatment enhanced the formation of the complex type of *N*-glycan more than the high mannose type. In conclusion, PRMT5 overexpression is related to the development of BLV infection with a high proviral load and lymphoma stage and PRMT5 inhibition enhances BLV gene expression. This is the first study to investigate the role of PRMT5 in BLV infection in vivo and in vitro and to reveal a novel function for a small-molecule compound in BLV-gp51 glycosylation processing.

## 1. Introduction

Bovine leukemia virus (BLV) is the causative agent of enzootic bovine leukosis, a B-cell leukemia and lymphoma [1,2], which occurs worldwide and causes serious losses in the cattle industry [3]. Approximately 70% of infected cattle, the natural host of BLV, remain asymptomatic, whereas the major portion of the remaining infected cattle develops persistent lymphocytosis (PL) and only 1–5% develop leukemia/lymphoma after a relatively long period of latency [1]. Therefore, BLV infection results not only in enzootic bovine leukosis development, but also in reductions in lifetime milk production, reproductive efficiency, and lifespan [3,4,5].

BLV belongs to the *Deltaretrovirus* genus of the Retroviridae family and is closely related to human T-cell leukemia viruses types 1 and 2 (HTLV-1 and -2) [1,2]. The HTLV-1 proviral load (PVL), which represents the retroviral genome integrated into the host genome, is an important risk factor of viruses-associated disease prediction [6]. Similarly, BLV PVL correlates strongly not only with the BLV infection capacity as assessed by syncytium formation [7,8], but also with BLV disease progression [7,9,10]. For example, BLV-infected cattle at the PL stage are known to carry a significantly increased number of PVL compared to the number of the PVL in aleukemic cattle. A further increase was observed at the lymphoma stage [7,9]. Additionally, BLV PVL is a useful index for estimating transmission risk [11]. A previous report demonstrated that cows with a PVL of greater than 14,000 copies/10^5^ cells and 18,000 copies/10^5^ cells in blood samples secreted BLV into the nasal mucus [12]. Furthermore, we recently determined that BLV provirus in infected cows was detected in milk samples only when the PVL in the blood samples exceeded 10,000 copies/10^5^ cells [13]. These findings suggest that a PVL of approximately 10,000 copies/10^5^ peripheral blood cells is a relatively high number and indicates BLV spreading within the whole body. Thus, a PVL of 10,000 copies/10^5^ cells was used in our study as a threshold to classify BLV-infected cattle into high-PVL (HPVL) or low-PVL (LPVL). In contrast, a low PVL (LPVL) is known to prevent natural BLV infection in cattle [14]. One of the host factors affecting BLV PVL is the genetic variation of bovine leukocyte antigen (*BoLA*). Several studies have identified *BoLA-DRB3* alleles and single-nucleotide polymorphisms in the *BoLA* region that are associated with BLV PVL and disease progression [9,15,16,17,18,19,20]. However, other factors contributing to the susceptibility to BLV-induced lymphoma and PVL, in addition to *BoLA-DRB3* polymorphism, remain to be identified.

In addition to the structural genes *gag*, *pro*, *pol*, and *env*, the BLV genome contains the regulatory genes *tax* and *rex* and genes R3 and G4 [1,2]. *gag* of BLV is translated as the precursor Pr70 Gag and is processed into three mature proteins: p15 is the matrix protein, p24 is the most abundant capsid protein, and p12 is the nucleocapsid protein [21,22]. *env* of BLV encodes a Pr72 envelope (Env) precursor that is glycosylated in the rough endoplasmic reticulum and Golgi apparatus [21,23]. This precursor is cleaved by cellular proteases into two mature proteins—the surface subunit gp51 and transmembrane subunit gp30—which are associated by disulfide bonds [21,24]. BLV infection is mediated by the interaction of gp51 with the recently identified cellular receptor cationic amino acid transporter 1 [25]. This gp51 is highly glycosylated because the molecular weight of its peptide backbone without glycans is 30.5 kDa and it contains eight asparagine (N) residues that are putative *N*-glycosylation sites [23]. BLV surface subunit gp51-linked *N*-glycosylation appeared to regulate viral replication, antigenic conformation, and syncytium forming capacity in vitro [26,27] and infectivity in vivo [26]. In general, retroviral Env glycosylation mediates virion attachment to cell membranes, transport of Env proteins to the cell membrane, and cell-to-cell fusion [28,29,30]. In contrast, Env-associated glycans act as a shield that confers resistance to neutralizing antibodies [30,31].

Arginine methylation is an important posttranslational modification with crucial roles in chromatin regulation, transcription control, RNA processing, nuclear/cytoplasmic shuttling, DNA repair, and other biological processes [32,33]. Arginine methylation is catalyzed by members of the protein arginine-*N*-methyltransferase (PRMT) family. PRMTs are divided into four types of enzymes. Type I, which is the most common, induces asymmetric dimethylation; Type II catalyzes symmetric dimethylation; Type III produces monomethyl arginine as its final product; and Type IV is found only in fungi [34]. In the virology field, protein arginine methylation has been determined to play critical roles in the biology of several viruses, including hepatitis delta virus [35], hepatitis B virus [36,37,38], human immunodeficiency virus type-1 (HIV-1) [39,40,41,42,43], HTLV-1 [44], Epstein-Barr virus (EBV) [45,46], and Kaposi’s sarcoma associated herpesvirus [47].

PRMT5 is a type II arginine methyltransferase that was recently shown to be crucial in EBV-driven B-cell transformation [46] and HTLV-1-mediated T-cell transformation [44]. Additionally, PRMT5 regulates various steps in virus lifecycles. For example, PRMT5 restricts hepatitis B virus replication via two mechanisms: epigenetic suppression of covalently closed circular DNA and interference with pregenomic RNA encapsidation [38]. Further studies showed that PRMT5 regulates nuclear import of hepatitis B virus core (HBc) in which PRMT5 overexpression increases the nuclear accumulation of HBc and in which PRMT5 inhibition reduces HBc levels in the nuclei [36]. Moreover, the early lytic protein of Kaposi’s sarcoma-associated herpesvirus, ORF59, associates with PRMT5 and disrupts its binding with the chromatin, which in turn disrupts its repressive effect to move to the lytic reactivation phase [47]. A recent study also demonstrated that PRMT5 supports HIV-1 replication by maintaining Vpr protein stability [48]. However, it remains unclear whether PRMT5 acts as a host cell factor that is important in BLV gene expression and infection.

In this study, we investigated the role of PRMT5 in various aspects of BLV infection in vivo and in vitro. First, we focused on the correlation between the PRMT5 expression level and BLV PVL, an index of virus infectivity, in peripheral blood from infected animals in various stages, such as the lymphoma and asymptomatic stages. Second, we revealed the impact of PRMT5 inhibition on BLV gene expression, gp51 glycosylation, and syncytium formation. This is the first study to investigate the role of PRMT5 in BLV infection.

## 2. Materials and Methods

### 2.1. Cell Culture and Transfection

FLK-BLV cells, which are permanently infected with BLV (kindly provided by Prof. Onuma, M.), were cultured in Dulbecco’s modified Eagle’s Medium (Thermo Fisher Scientific, Waltham, MA, USA) containing 10% heat-inactivated fetal bovine serum (Sigma-Aldrich, St. Louis, MO, USA). PK15-BLV cells, which were produced by stably transfecting the pig kidney-15 (PK15) cells (National Institutes of Biomedical Innovation, Health and Nutrition: JCRB9040) with CMV∆U3-pBLV-IF2, were cultured in Minimum Essential Medium Eagle (Thermo Fisher Scientific) containing 10% fetal bovine serum and 1% non-essential amino acids (Gibco, Grand Island, NY, USA). CMV∆U3-pBLV-IF2 is the modified version of the BLV-infectious molecular clone pBLV-IF2, which was used previously [25,49]. CMV∆U3-pBLV-IF2 was modified by replacing the U3 region that contains the BLV promoter with the strong CMV promoter to enhance BLV expression. FLK-BLV and PK15-BLV were selected because they stably express BLV-viral proteins and produce BLV-virus particles, enabling analysis of several aspects of the virus life cycle related to the expression of viral proteins.

For transfection of small interfering RNA (siRNA), we used Lipofectamine RNAiMax Reagent (Thermo Fisher Scientific) according to the manufacturers’ instructions and RNA was extracted from these cells using TRIzol reagent (Thermo Fisher Scientific) according to the manufacturer’s protocol.

### 2.2. Animal Samples and Isolation of Genomic DNA and RNA

Blood samples were collected from 62 Holstein cattle including BLV-negative cattle and BLV-infected cattle, which were maintained in Japan. These cows were classified into three groups according to their PVL as follows: (i) BLV-negative cattle (PVL = 0; *N* = 20), (ii) BLV-infected cattle with LPVL (PVL ≤10,000 copies/10^5^ cells; *N* = 15), and (iii) BLV-infected cattle with HPVL (PVL >10,000 copies/10^5^ cells; *N* = 27). Blood samples were also collected from BLV-infected Holstein cows with lymphoma (*N* = 20). Lymphoma was diagnosed by both gross and histological observation and by detecting atypical mononuclear cells in the slaughterhouse. All animal experiments were conducted in accordance with the guidelines for Laboratory Animal Welfare and Animal Experiment Control established by the RIKEN Animal Experiments Committee (H29–2-104, 14 February 2017). For experimental infection, five BLV-negative one-year-old Japanese black calves carrying susceptible alleles BoLA-DRB3*1601/*1601 were experimentally challenged intravenously with blood containing a PVL of 4 × 10^7^ copies/10^5^ cells. This study was approved by the Animal Ethical Committee and Animal Care and Use Committee of the Kyoto Biken Institute.

Blood samples were collected and diluted in a 1:1 ratio with Ambion^®^ nuclease-free water before adding TRIzol LS in a 3:1 ratio of TRIzol LS to the diluted whole blood. The mixtures of whole blood with TRIzol LS were transported to the lab and stored at −80°C. An equal volume of the mixture was used to extract RNA from the samples using TRIzol LS protocol (Thermo Fisher Scientific) according to the manufacturer’s instructions. Genomic DNA for PCR amplification was isolated from EDTA-treated whole blood samples using the Wizard Genomic DNA Purification Kit (Promega Corporation, Madison, WI, USA).

### 2.3. Measurement of the BLV PVL

The BLV PVL was measured by BLV-CoCoMo-qPCR-2 (RIKEN Genesis, Kanagawa, Japan) using genomic DNA as described previously [50]. Briefly, the BLV long terminal repeat (LTR) region was amplified in a reaction mixture containing THUNDERBIRD Probe qPCR Mix (Toyobo, Osaka, Japan) and the degenerate primer pair: CoCoMo FRW primer and CoCoMo REV primer. FAM-LTR was used as a probe. *BoLA-DRA* (internal control) was amplified using the primer pair DRA-F and DRA-R. FAM-DRA was used as a probe. The PVL was calculated using the following equation: (number of BLV-LTR copies/number of *BoLA-DRA* copies) × 10^5^ cells.

### 2.4. Quantitative Reverse Transcription–Polymerase Chain Reaction (qRT-PCR)

RNA was reverse-transcribed for cDNA synthesis using a High Capacity RNA-to-cDNA kit (Thermo Fisher Scientific) according to the manufacturer’s instructions. Primers for PRMT5, GAPDH, *gag*, and *tax* were designed using the primer-designing tool provided by the National Center for Biotechnology Information. The primer list is shown in Table S1. RT-PCR was performed with the KAPA SYBR^®^ FAST qPCR Kit (KAPA BIOSYSTEMS, Wilmington, MA, USA) using an Applied Biosystems 7500 Fast Real-Time PCR system (Foster City, CA, USA). The following thermal cycling program was used: 95 °C for 3 min, followed by 40 cycles of 95 °C for 10 s and 60 °C for 30 s. Samples were evaluated in duplicate and data analysis was performed using the comparative CT method (∆∆CT) with normalization to GAPDH mRNA expression.

### 2.5. siRNA Transfection

siRNA designed to target PRMT5 (si-PRMT5) of FLK-BLV or PK15-BLV was constructed by Silencer Select siRNAs (Ambion, Austin, TX, USA). To knock down PRMT5 in FLK-BLV cells, Silencer^®^ select Product # s20376 was used. For PK15-BLV knockdown, Silencer^®^ select Product # s20377 was used. siRNA negative control (si-NC) was constructed by Ambion (catalog# 4390843). On the day of transfection, 3 × 10^5^ cells of FLK-BLV or PK15-BLV were seeded into a 6-well plate and transfected with si-PRMT5 or si-NC using Lipofectamine RNAiMAX reagent (Thermo Fisher Scientific) according to the manufacturer’s instructions. After 48 or 72 h of incubation, cell lysates were prepared and knockdown efficiency was evaluated by RT-PCR or Western blotting analysis.

### 2.6. PRMT5 Inhibitor Treatment

CMP5, a selective PRMT5 inhibitor [46], was purchased from Merck KGaA (Darmstadt, Germany). Cells were grown for 24 h at 37 °C, after which the medium was collected and replaced with fresh medium with or without 5, 10, or 20 µM of CMP5 and the cells were grown for an additional 24 h. After a total of 48 h, cell lysates were prepared to determine intracellular expression of PRMT5 by Western blotting analysis.

### 2.7. Western Blotting Analysis

Cells were lysed for 30 min on ice in 20 mM Tris-HCl (pH 7.4), 300 mM NaCl, 2 mM EDTA, and 2% NP40 supplemented with a protease inhibitor cocktail (Roche Diagnostics, Mannheim, Germany). Lysates were mixed with SDS buffer and boiled for 5 min. Protein concentrations were determined with Pierce™ BCA Protein Assay Kit (Thermo Fisher Scientific). Equal amounts of protein were electrophoresed via SDS-polyacrylamide gel electrophoresis (PAGE). The proteins were then transferred onto a polyvinylidene difluoride membrane (Millipore, Billerica, MA, USA) using a Trans-Blot Turbo apparatus (Bio-Rad, Hercules, CA, USA) and incubated with anti-BLV gp51 monoclonal antibody (Mab) (BLV 2) (1:200; VMRD, Pullman, WA, USA), anti-BLV Gag Mab (BLV 3) (1:200; VMRD), polyclonal anti-PRMT5 (1:1000; Abcam, Cambridge, UK), or anti-α-tubulin clone B-5-1-2 Mab (1:2000; Sigma). After washing, the membranes were incubated with horseradish peroxidase-conjugated AffiniPure goat anti-mouse IgG (1:2000; Jackson ImmunoResearch, West Grove, PA, USA) or horseradish peroxidase-conjugated goat anti-rabbit IgG (1:1000; Amersham Biosciences, Amersham, UK). Signals were visualized after treating the membrane with SuperSignal™ West Pico PLUS Chemiluminescent Substrate (Thermo Fisher Scientific). Images were acquired using a MultiImage TM light Cabinet (Alpha Innotech Corporation, San Leandro, CA, USA). Band intensity was quantitated using the analysis tool provided with the AlphaEaseFC^TM^ software (Alpha Innotech Corporation).

### 2.8. Deglycosylation by PNGase F or Endo H

FLK-BLV or PK15-BLV were cultured in the absence or presence of 20 µM CMP5 for 48 h. Thereafter, the cell lysates were treated in the absence or presence of PNGase F (Promega, Madison, WI 53711-5399, USA) for 3 h or Endo H (Promega) overnight according to the manufacturer’s protocol (catalog# V483A and catalog #V4871), respectively.

### 2.9. Cell Viability Assay

A Premix WST-1 Cell Proliferation Assay Kit (Takara, Shiga, Japan) was used to assess the effect of CMP5 on cell viability. For each cell line, 3 × 10^4^ cells were seeded into a 96-well plate at a final volume of 100 µL in the absence or presence of different concentrations of CMP5. The assay was conducted according to the manufacturer’s instructions.

### 2.10. Immunofluorescence Confocal Microscopy

FLK-BLV cells were grown on coverslips with or without CMP5 treatment. After 48 h, the cells were fixed with 4% paraformaldehyde for 15 min at room temperature; next, they were permeabilized with 0.5% Triton X for 5 min and then blocked with 5% skim milk for 30 min. The gp51 staining in the cell membrane was performed without the permeabilization step. The cells were incubated with anti-BLV gp51 Mab (BLV 2) (1:100; VMRD) or anti-BLV Gag Mab (BLV 3) (1:100; VMRD) for 1 h at room temperature followed by 30 min incubation with Alexa Fluor 488 rabbit anti-mouse for Gag or Alexa Fluor 594 rabbit anti-mouse for Env (1:300; Invitrogen, Carlsbad, CA, USA). Nuclei were stained with Hoechst 33342 (1:2000; ImmunoChemistry Technologies LLC. Bloomington, MN, USA) for 5 min in the dark. After each staining step, the samples were washed three times with PBS. The coverslips were mounted on glass slides and fluorescence images were obtained using an FV1000 confocal laser-scanning microscope (Olympus, Tokyo, Japan).

### 2.11. gp51 Cell Membrane Staining by Flow Cytometry

FLK-BLV cells were collected using 2 mM ethylenediaminetetraacetic acid (EDTA)/PBS for 5 min at 37 °C and washed twice with PBS and 5 × 10^5^ cells were stained with anti-BLV gp51 Mab (BLV 2) (1:50; VMRD) for 1 h on ice. The cells were then stained with APC rat anti-mouse (1:100; APC-anti mouse, BD Pharmingen, Franklin Lakes, NJ, USA) for 30 min on ice. Propidium Iodide (PI) was used for live/dead staining. Stained cells were analyzed using a BD AccuriTM C6 Plus with a sampler flow cytometer (BD Biosciences, Franklin Lakes, NJ, USA). The data were analyzed using FlowJo v10 (FlowJo, LLC, Ashland, OR, USA).

### 2.12. Luminescence Syncytium Induction Assay (LuSIA)

The luminescence syncytium induction assay (LuSIA) using CC81-GREMG cells was performed as described previously [51]. CC81 GREMG (1 × 10^5^ cells) were co-cultured with 5 × 10^4^ FLK-BLV cells in a 12-well plate in LuSIA medium, after which the indicated concentrations of CMP5 were added. After 48 h incubation, the cells were washed with PBS and fixed with 4% formaldehyde/PBS containing 10 µg/mL Hoechst 33342 (Thermo Fisher Scientific). The fluorescent syncytia were visualized by EVOS2 fluorescence microscopy (Thermo Fisher Scientific) and 9 fields of view in each well were automatically scanned with a 4× objective. Syncytia were detected by enhanced green fluorescent protein (EGFP) expression and counted using HCS Studio Cell Analysis software (Thermo Fisher Scientific).

### 2.13. Statistical Analysis

All data were expressed as the mean ± standard deviation based on at least 3 independent experiments. Statistical significance was evaluated using Student’s *t*-test. Differences were estimated to be significant at *p* ≤ 0.05 (*) and strongly significant at *p* ≤ 0.01 (**) and *p* ≤ 0.001(***). The *p* value in the animal experiments was calculated by the Kruskal-Wallis test and Dunn’s multiple comparisons test.

## 3. Results

### 3.1. PRMT5 Is Overexpressed in BLV-Infected Cattle with High *PVL* In Vivo

**Figure 1:**
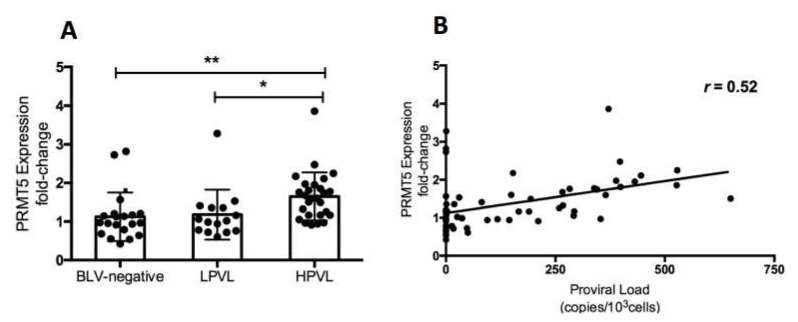
Change in PRMT5 expression at the RNA level in bovine leukemia virus (BLV) infection. (**A**) PRMT5 expression at the RNA level of 20 BLV-negative cattle (PVL, proviral load = 0), 15 BLV-infected but clinically normal cows with a low PVL (LPVL; PVL ≤ 10,000 copies/10^5^ cells), and 27 BLV-infected but clinically normal cows with a high PVL (HPVL; PVL > 10,000 copies/10^5^ cells) was measured by qRT-PCR and the fold-change was calculated using the ΔΔCT method with normalization to GAPDH expression as an internal control. Samples were run in duplicate. A scatter plot with bar showing the mean of PRMT5 expression fold-change in the three groups. BLV PVL was measured by CoCoMo-qPCR. Error bars represent the standard deviation of the biological replicates (20 BLV-negative, 15 LPVL, 27 HPLV). The *p* value was calculated using the Kruskal-Wallis test (<0.0006). Dunn’s multiple comparisons test was used to evaluate the significance between groups. The asterisk indicates a significant difference (* *p* ≤ 0.05, ** *p* ≤ 0.01). (**B**) Correlation between BLV PVL of each cattle and the corresponding PRMT5 expression fold-change (62 in total), Spearman *r* was used to evaluate the strength of the correlation.

BLV PVL is an important index of the risk of BLV transmission and infectivity as well as disease progression [7,8,10,12,13]. To investigate the correlation between PRMT5 expression and BLV PVL, we collected blood samples from 62 cows which were asymptomatic and had not developed symptoms of lymphoma at the time of blood collection. First, we performed CoCoMo-qPCR-2 to calculate the BLV PVL, which was used to classify the cows into three groups: BLV-negative cattle (control group), BLV-infected cattle with a low-PVL (LPVL group), and BLV-infected cattle with a high-PVL (HPVL group). As described previously [9,15], a BLV-PVL of 10,000 copies/10^5^ was used as a threshold to distinguish between the HPVL and LPVL groups. Next, we evaluated PRMT5 expression at the RNA levels by qRT-PCR. The mean fold-change in PRMT5 was 1.12 ± 0.62 in the control group, 1.18 ± 0.64 in the LPVL group, and 1.64 ± 0.62 in the HPVL group (Figure 1A). PRMT5 expression was significantly higher in the HPVL group than in the uninfected group (*p* = 0.0014) and LPVL group (*p* = 0.012) (Figure 1). Our results showed that PRMT5 was significantly overexpressed only in BLV-infected cattle with a high PVL, but not in those with a low PVL. Furthermore, a positive correlation was obtained between the BLV PVL and PRMT5 expression fold-change (*r* = 0.52) (Figure 1B).

### 3.2. PRMT5 Overexpression Starts from an Early Stage of BLV Infection In Vivo

**Figure 2:**
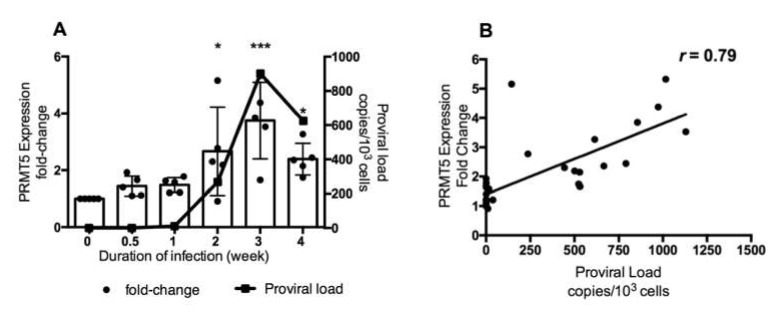
Change in PRMT5 expression at the RNA level in response to experimental BLV infection during the early stage of infection. (**A**) Five BLV-negative Japanese black calves carrying susceptible alleles BoLA-DRB3*1601/*1601 were experimentally challenged intravenously with BLV, and RNA and DNA were extracted from these animals at six time points during the first month of infection. The BLV PVL was measured by CoCoMo-qPCR using genomic DNA. PRMT5 expression was measured by qRT-PCR using RNA and the fold-change was calculated using the ΔΔCT method with normalization to GAPDH expression as an internal control. Samples were run in duplicate. A combined bar and line graph show the mean of PRMT5 expression fold-change at each point (bar graph and left axis) with the corresponding mean of proviral load (line graph and right axis). Error bars represent the standard deviation of the PRMT5 fold-change of five cattle, *p* value was calculated using the Kruskal-Wallis test (0.002), and Dunn’s multiple comparisons test was used to compare the mean of the PRMT5 fold-change at each time point of infection with the mean before infection. The asterisk indicates a significant difference (* *p* ≤ 0.05 and *** *p* ≤ 0.001). (**B**) Correlation between BLV PVL of each cattle at each time point and the corresponding PRMT5 expression fold-change, Spearman *r* was used to evaluate the strength of the correlation.

As shown in Figure 1, PRMT5 was upregulated only in BLV-infected cattle with a high PVL. To further investigate the role of PRMT5 in BLV infection in vivo, we examined whether PRMT5 is overexpressed in the early stage of BLV infection. Therefore, we performed experimental infection of five BLV-negative Japanese black calves carrying the susceptible alleles BoLA-DRB3*1601/1601, which are related to a high PVL in BLV infection [52] and we collected blood samples at different time points during the first month of infection (0, 0.5, 1, 2, 3, and 4 weeks). Next, we monitored BLV PVL and PRMT5 expression at each time point. The mean of BLV PVL was 0 copies/10^5^ cells before infection (0 week), 34 copies/10^5^ cells after 3 days, 1158 copies/10^5^ cells after 1 week, 26,760 copies/10^5^ cells after 2 weeks, 90,150 copies/10^5^ cells after 3 weeks, and 62,440 copies/10^5^ cells after 4 weeks (Figure 2a, line graph). The mean fold-change in PRMT5 was 1 before infection, 1.44 after 3 days, 1.48 after 1 week, 2.6 after 2 weeks, 3.7 after 3 weeks, and 2.3 after 4 weeks. The highest expression of PRMT5 was observed during the third week (fold-change = 3.74, *p* = 0.0008) and then the expression slightly decreased after 4 weeks (fold-change = 2.3, *p* = 0.01) (Figure 2A, bar graph). Interestingly, and consistently with the data shown in Figure 1A, a strong positive correlation was found between the fold-upregulation of PRMT5 and BLV PVL (*r* = 0.79) (Figure 2B). Thus, PRMT5 expression was upregulated in response to BLV infection and the level of upregulation was positively correlated with the BLV PVL. Moreover, PRMT5 upregulation began in an early stage of BLV infection rather than being established after a long period of proviral latency.

### 3.3. PRMT5 Overexpression Continues to Lymphoma Stage of BLV Infection In Vivo

**Figure 3:**
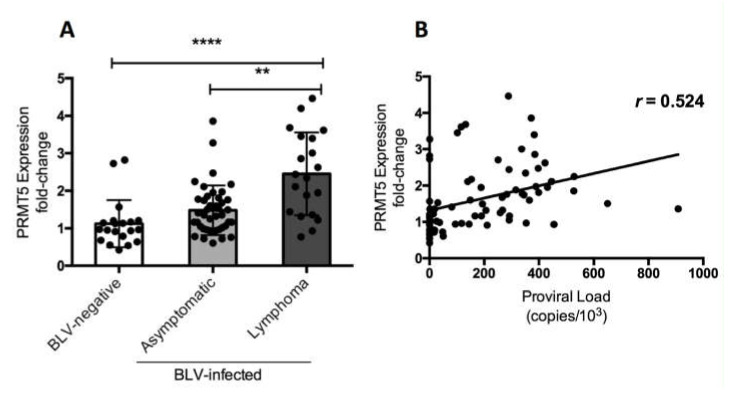
PRMT5 gene expression at the RNA level in BLV-infected cattle with lymphoma. (**A**) PRMT5 expression at the RNA level of 20 BLV-negative cattle, 42 BLV-infected but clinically normal cows, and 20 BLV-infected cattle with lymphoma were measured by qRT-PCR and the fold-change was calculated using the ΔΔCT method with normalization to GAPDH expression as an internal control. Samples were run in duplicate. A scatter plot with a bar showing the mean of the fold-change in PRMT5 expression of the three groups of cattle. BLV PVL was measured by CoCoMo-qPCR. The *p* value was calculated using the Kruskal-Wallis test (<0.0001) and Dunn’s multiple comparisons test was used to evaluate the significance among groups. The asterisk indicates a significant difference (** *p* ≤ 0.001 and **** *p* ≤ 0.0001). (**B**) Correlation between BLV PVL of each cow and corresponding PRMT5 expression fold-change (81 in total), Spearman *r* was used to evaluate the strength of the correlation.

Finally, we investigated whether the change in PRMT5 expression in BLV-infected cattle occurred in the lymphoma stage of disease. We compared PRMT5 expression at the RNA level among three groups of cattle: 20 BLV-negative cattle, 42 BLV-infected but clinically normal cattle, and 20 BLV-infected cattle with lymphoma. The fold-change in PRMT5 expression was 1.12 ± 0.62 in BLV-negative cattle, 1.48 ± 0.66 in the asymptomatic group, and 2.45 ± 1.1 in the lymphoma group (Figure 3). This strongly indicates that PRMT5 upregulation continues until the lymphoma stage of BLV. Additionally, we confirmed the positive correlation between BLV PVL and PRMT5 expression fold-change (*r* = 0.524).

### 3.4. PRMT5 Knockdown Enhances BLV Gene Transcription In Vitro

**Figure 4:**
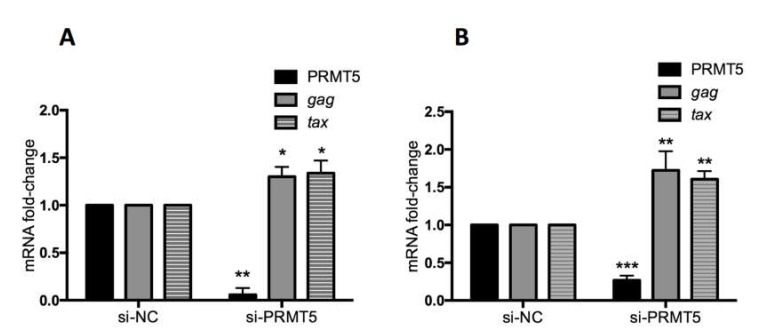
Effect of PRMT5 knockdown on BLV gene transcription. FLK-BLV (**A**) or PK15-BLV (**B**) cells were transfected with scramble siRNA (si-NC) or siRNA targeting PRMT5 (si-PRMT5) for 48 h for FLK-BLV or 72 h for PK15-BLV. BLV gene transcription (mRNA-*gag* and mRNA-*tax*) in addition to PRMT5 gene expression at the RNA level was evaluated by qRT-PCR. The ΔΔCT method was used to calculate the fold-change and GAPDH expression was used as an internal control. Error bars represent the standard deviation of three experiments. The *p* value was calculated using the Student’s *t*-test. The asterisk indicates a significant difference (* *p* ≤ 0.05, ** *p* ≤ 0.01, and *** *p* ≤ 0.001).

The data shown in Figure 1, Figure 2 and Figure 3 indicate that PRMT5 overexpression contributes to developing BLV infection with a high proviral load and may influence which infected cows progress from the asymptomatic stage to the lymphoma stage. Next, we examined the impact of PRMT5 inhibition on BLV infection in vitro. PRMT5 is a well-known regulator of gene transcription either by catalyzing symmetric dimethylarginine of histone proteins to generate repressive histone markers, including H2AR3me2s, H3R8me2s, and H4R3me2s, or by methylation of nonhistone proteins, such as transcription factors [33,53]. To investigate the effect of PRMT5 inhibition on BLV gene expression, we knocked down PRMT5 in two cell lines, FLK-BLV, a permanently BLV-infected cell line, and PK15-BLV, a stably transfected cell line with CMV∆U3-pBLV-IF2, by siRNA. Next, we measured the mRNA of two viral transcripts, *gag*, which is produced by the un-spliced mRNA, and *tax*, which is produced by the double splicing event (Figure 4). We found that PRMT5 knockdown significantly enhanced the *gag* and *tax* transcription levels. In the FLK-BLV cell line, the fold-change of *gag* was 1.3 (*p* = 0.03) and of *tax* was 1.33 (*p* = 0.04). In the PK15-BLV cell line, the fold-change of *gag* was 1.72 (*p* = 0.01) and of *tax* was 1.6 (*p* = 0.0014).

### 3.5. PRMT5 Knockdown Enhances BLV Protein Expression In Vitro

**Figure 5:**
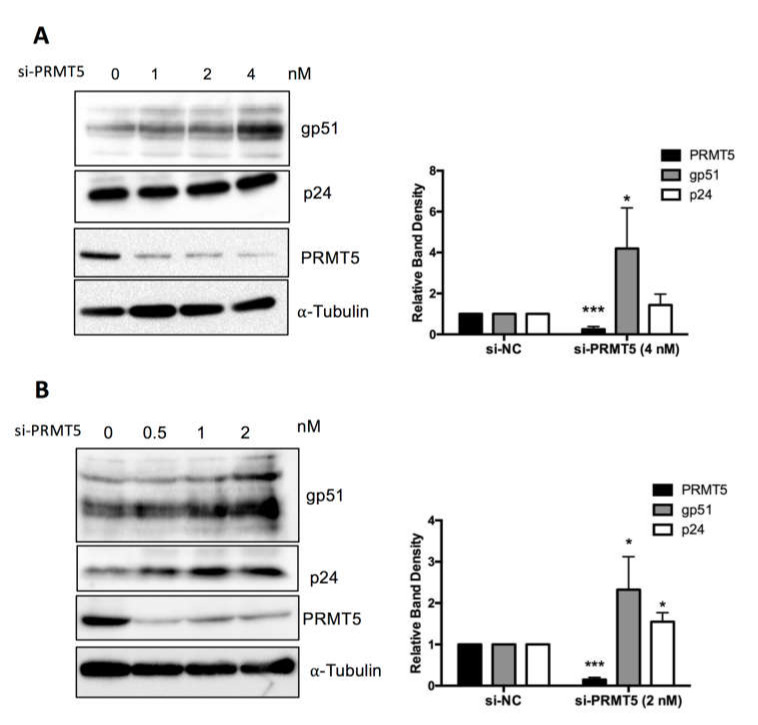
Effect of PRMT5 knockdown on BLV protein expression. FLK-BLV (**A**) or PK15-BLV (**B**) cells were transfected with scramble siRNA (si-NC) or siRNA targeting PRMT5 (si-PRMT5) at the indicated concentrations for 48 h for FLK-BLV or 72 h for PK15-BLV. Cell lysates were prepared and the expression of two viral proteins (Env gp51 and Gag p24) were assessed by Western blotting. Knockdown efficiency was evaluated by evaluating PRMT5 protein expression by Western blotting. The relative band density of each protein normalized to α-tubulin expression is shown for each cell line. Error bars represent the standard deviation of three experiments. The *p* value was calculated by Student’s *t*-test. The asterisk indicates a significant difference (* *p* ≤ 0.05, and *** *p* ≤ 0.001). Positions of BLV gp51 and p24, PRMT5, and α-tubulin proteins are indicated.

To confirm the finding shown in Figure 4 at the protein level, FLK-BLV or PK15-BLV were transfected with siRNA targeting PRMT5 or mock siRNA for 48 or 72 h and then cell lysates were prepared and the expression levels of two viral proteins (Env gp51and Gag p24) were evaluated by Western blotting with anti-BLV gp51 Mab or anti-BLV p24 Mab. We confirmed that siRNA-mediated inhibition of PRMT5 in FLK-BLV significantly enhanced intracellular Env expression (*p* = 0.02) (Figure 5A). Moreover, the expression of both of Env and Gag in PK15-BLV were significantly enhanced as a result of PRMT5 knockdown (*p* = 0.05 and 0.02 for Env and Gag, respectively) (Figure 5B). The bands corresponding to unprocessed Gag are shown in Figure S2A,B for FLK-BLV and PK15-BLV, respectively. These results show that there was no defect in Gag processing after PRMT5 knockdown.

### 3.6. Selective PRMT5 Inhibitor Enhances BLV Protein Expression and Alters gp51 Mobility over SDS-PAGE In Vitro

**Figure 6:**
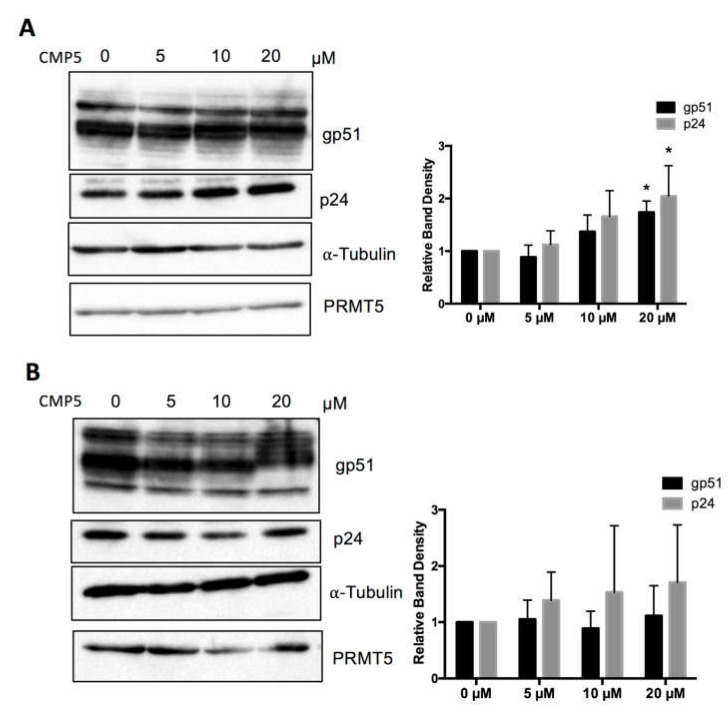
Effect of a small molecular PRMT5 inhibitor (CMP5) on BLV protein expression. FLK-BLV (**A**) or PK15-BLV (**B**) were cultured in the absence or the presence of the indicated concentrations of CMP5 for 48 h. Cell lysates were prepared and viral proteins (Env gp51 and Gag p24) in addition to PRMT5 were evaluated by Western blotting. The relative band density of each protein normalized to α-tubulin expression is shown for each cell line. The PRMT5 expression level was not changed as a result of CMP5 treatment and was excluded from analysis. All band layers of gp51 were calculated. Error bars represent the standard deviation of three independent experiments. The *p* value was calculated using the Student’s *t*-test. The asterisk indicates a significant difference (* *p* ≤ 0.05). Positions of BLV gp51 and p24, PRMT5, and α-tubulin proteins are indicated.

Next, we examined the effect of a pharmacological PRMT5 inhibitor on BLV protein expression. We used a small-molecule PRMT5 inhibitor (CMP5), which was described previously by Alinari et al. [46]. We examined the effect of CMP5 on FLK-BLV and PK15-BLV. FLK-BLV or PK15-BLV was cultured in fresh medium with or without 5, 10, or 20 µM of CMP5 for 48 h. Cell lysates were prepared and the viral protein expression levels of Env and Gag were evaluated by Western blotting analysis with anti-BLV gp51 Mab or anti-BLV p24 Mab. Consistently, CMP5 treatment enhanced the protein expression of both gp51 and p24 in a dose-dependent manner in FLK-BLV, showing significance at a concentration of 20 µM (Figure 6A). In contrast, in PK15-BLV, BLV expression was not significantly affected (Figure 6B). The bands corresponding to unprocessed Gag are shown in Figure S2C,D for FLK-BLV and PK15-BLV, respectively. These results also revealed that there was no defect in Gag processing after CMP5 treatment.

Unexpectedly, we found that CMP5 treatment altered gp51 electrophoretic mobility in SDS-PAGE and formed gp51 with a higher apparent molecular weight than gp51 in untreated cells (Figure 6A,B). The change in the apparent molecular weight was more evident at 20 µM. Thus, we further investigated the possible impact of this inhibitor on BLV envelope protein glycosylation.

### 3.7. Selective PRMT5 Inhibitor Alters gp51 Glycosylation Processing In Vitro

**Figure 7:**
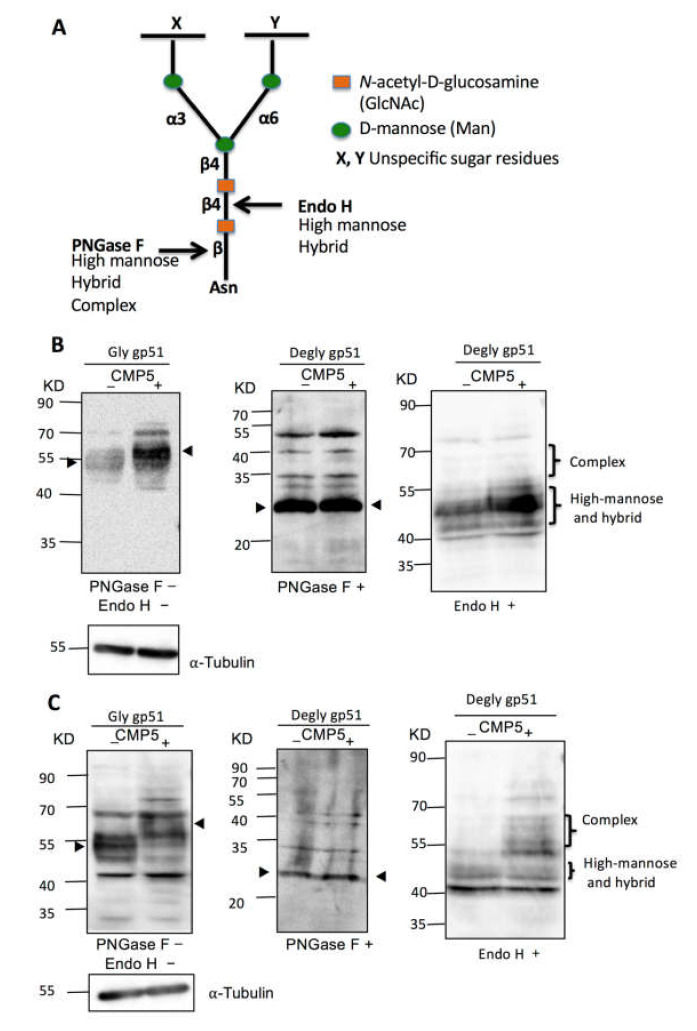
Deglycosylation assay of BLV gp51 using PNGase F and endo H. (**A**) Schematic representation of PNGase F and Endo H sensitive bonds in the core of *N*-glycans. (**B**) FLK-BLV or (**C**) PK15-BLV were treated with Milli-Q water (CMP5−) or 20 µM CMP5 (CMP5+) for 48 h. Cell lysates were divided into three parts: one part was untreated (PNGase F−/Endo H−), the second part was deglycosylated by PNGase F (PNGase F+), and the third part was deglycosylated by Endo H (Endo H+). The cell lysates from the three conditions were subjected to Western blotting analysis with anti BLV gp51 (BLV2). α-Tubulin was used as a loading control. The data are a representation of at least three experiments. The positions of BLV gp51 protein with or without glycosylation, gp51 glycosylation pattern such as complex, and high-mannose and hybrid pattern, in addition to α-tubulin protein, are indicated.

*N*-Glycan structures are classified into three types: high mannose, hybrid, and complex types. All types share a common tri-mannosyl core (Man3GlcNAc2). However, they differ in the structure of the remaining branches [54,55]. The complex type of *N*-glycan typically runs slower in SDS-PAGE than the high mannose type. To further illustrate the effect of CMP5 treatment on gp51 electrophoretic mobility in SDS-PAGE, we examined whether the slow migration of gp51 in the presence of CMP5 resulted from an increase in the complex type of *N*-glycan. We cultured FLK-BLV or PK15-BLV in the absence (CMP5–) or presence of 20 µM of CMP5 (CMP5+) for 48 h. Cell lysates of each cell line were collected and divided into three parts: one part was not treated (PNGase F−/Endo H−), the second part was treated with PNGase F (PNGase F+), and the third part was treated with Endo H (Endo H+). As illustrated in Figure 7A, PNGase F removed all types of *N*-linked glycosylation from the glycoprotein: high mannose, hybrid, and complex. Additionally, PNGase F severs the bond between *N*-acetylglucosamine (GlcNAc) and asparagine (Asn), liberating the entire sugar chain and converting asparagine (Asn) to aspartic acid (Asp). In contrast, Endo H cleaves only the high mannose and hybrid types of *N*-glycans and the complex type remains resistant to Endo H deglycosylation. Endo H cleaves the bond between the two GlcNAc residues in the core region, leaving one GlcNAc bound to the protein [56].

Consistent with the observation shown in Figure 6, we found that CMP5 treatment (CMP5+) altered the electrophoretic mobility of gp51, forming a higher apparent molecular weight smear of gp51 in both FLK-BLV and PK15-BLV (Figure 7B,C, PNGase F−/Endo H−). PNGase F treatment led to the accumulation of an approximately 30 KDa product in both FLK-BLV and PK15-BLV (Figure 7B,C, PNGase F+). This corresponds to the calculated molecular weight of the envelope peptidic core in the absence of any glycosylation of BLV gp51. Thus, after PNGase F treatment, the change in the migration pattern of gp51 disappeared, indicating that CMP5 affects the gp51 glycosylation pattern. Additionally, endo H treatment in PK15-BLV showed that the complex type of *N-*glycan of gp51 may increase after CMP5 treatment (Figure 7C, Endo H+), whereas in FLK-BLV, the complex type did not appear to be affected (Figure 7B, Endo H+). Our results show that CMP5 treatment altered gp51 glycosylation processing.

### 3.8. CMP5 Treatment Does Not Change Env Localization at the Plasma Membrane

**Figure 8:**
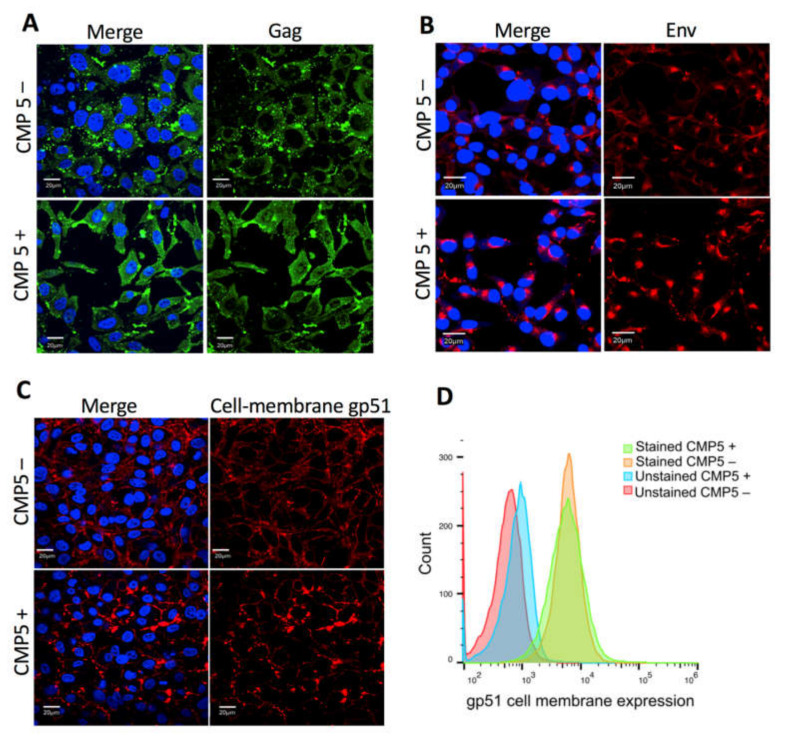
Effect of CMP5 on Gag and Env localization. (**A**,**B**) FLK-BLV were grown on coverslips and treated with Milli-Q water (CMP5–) or CMP5 20 µM (CMP5+), Gag was stained with green fluorescence (**A**), Env was stained with red fluorescence (**B**), The cellular membrane staining of gp51 was performed without the permeabilization step (**C**). DAPI (blue fluorescence) was used to stain the nucleus and the merge picture represents Gag or Env with DAPI. The data are a representation of three experiments. Images were acquired with a 60X objective and the scale bar is equal to 20 µm. (**D**) FLK-BLV were treated with Milli-Q water (CMP5–) or CMP5 20 µM (CMP5+), the cells were collected, and gp51 cell membrane expression was assessed by flow cytometry. The data are a representation of two experiments. Scale bar—20 µm.

Next, we examined whether CMP5 treatment affects Gag or Env localization or Env trafficking to the cell membrane. FLK-BLV cells were grown on coverslips in the absence (CMP5–) or presence of 20 µM of CMP5 (CMP5+) for 48 h; Gag was stained with a green fluorescent marker, Env was stained with a red fluorescent marker, and DAPI was used to stain the nucleus. Gag and Env localization were evaluated by fluorescence confocal microscopy. The cellular membrane staining of gp51was performed without the permeabilization step and the intracellular staining was conducted after the permeabilization with 0.5% Triton X-100 for 5 min. We observed a large accumulation of Gag after CMP5 treatment (Figure 8A). Additionally, the intracellular Env protein accumulated near to the nucleus after the treatment (Figure 8B). In contrast, the cell membrane expression of gp51 was not severely affected, as demonstrated by the fluorescence confocal microscopy (Figure 8C) and by flow cytometry (Figure 8D).

### 3.9. Selective PRMT5 Inhibitor Impedes BLV ENV-Mediated Syncytia formation In Vitro

Previous studies showed that perturbation of the *N*-linked glycan structure of the HIV and HTLV-1 envelope proteins affects syncytia formation [57,58]. Therefore, we examined the impact of the PRMT5 inhibitor CMP5 on the syncytium formation ability. We used LuSIA to quantify the syncytia [8,51]. Because examining the syncytia formation ability of PK15-BLV cells was not possible because of the relatively longer time required for forming the syncytia, we only assessed the effect of CMP5 on syncytia formation using the FLK-BLV cell line. The LuSIA depends on a new reporter cell line (CC81-GREMG) stably transfected with a reporter plasmid in which the EGFP reporter gene is expressed under control of the glucocorticoid response element (GRE)-mutated long terminal repeat (LTR)-U3 promoter and enables direct visualization of syncytia. CC81-GREMG expresses EGFP in response to BLV trans-activator p38tax expression and forms fluorescent syncytia when cultured with BLV-producing cells.

**Figure 9:**
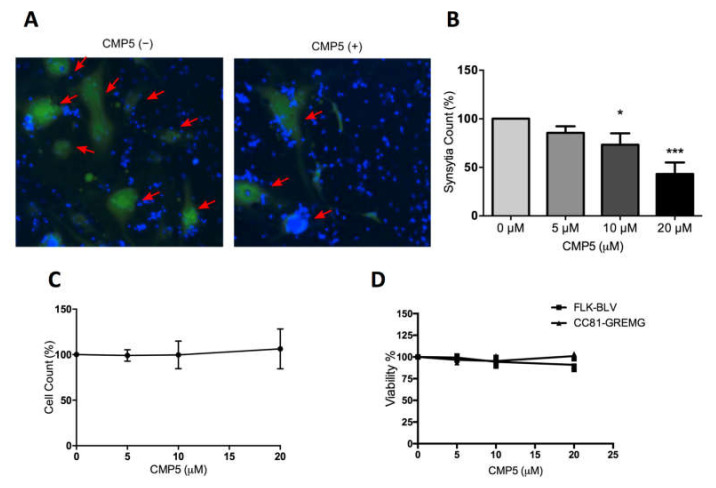
CMP5 effect on BLV ENV-mediated syncytia formation. A BLV-producing cell line FLK-BLV was co-cultured with a reporter cell line CC81-GREMG and treated with Milli-Q water (CMP5−) or CMP5 at the indicated concentrations (CMP5+) for 48 h. (**A**,**B**). Fluorescent syncytia in nine fields of view in each well were automatically scanned by EVOS2 fluorescence microscopy with a 4× objective. Fluorescent syncytia were recognized by enhanced green fluorescent protein (EGFP) expression and gated by their area and intensity. Red arrows indicate positions of fluorescent syncytia. Hoechst 33342 was used to stain the nucleus. Each picture is a representation of nine pictures acquired for each well. (**B**) Data analysis of (**A**) shows syncytia count using HCS Studio Cell Analysis software. Error bars represent the standard deviation of three independent experiments, the *p* value was calculated by ANOVA (0.00031), and Dunnett’s multiple comparisons test showed a significant decrease in the syncytia count at 10 µM (* *p* = 0.017) and 20 µM (*** *p* = 0.0002). (**C**) Effects of CMP5 on the total cell count taken from gating and counting of Hoechst 33342 staining using HCS Studio Cell Analysis software (Thermo Fisher Scientific). (**D**) CMP5 effect on cell viability according to WST-1 assay.

FLK-BLV cells were co-cultured with CC81-GREMG cell in the absence or presence of different concentrations of CMP5. Interestingly, CMP5 treatment negatively affected syncytia formation; this was detected by fluorescence microscopy (Figure 9A) and automated quantification showing that the syncytia counts were decreased in a dose-dependent manner, showing significance at concentrations of 10 µM (*p* = 0.017) and 20 µM (*p* = 0.0002) (Figure 9B). In contrast, the total cell count during the assay remained unaffected (Figure 9C). In addition, the maximum used concentration of CMP5 (20 µM) did not severely affect the viability of FLK-BLV or CC81-GREMG (Figure 9D). These observations exclude the toxic effect of CMP5 on FLK-BLV or CC81-GREMG. Our results obtained by LuSIA using CC81-GREMG cells strongly suggest that PRMT5 regulates the induction of the syncytia by BLV Env.

## 4. Discussion

BLV-mediated pathogenicity depends on a delicate balance between viral gene expression and certain host-related genetic and epigenetic events [2]. The mutation rate of BLV is relatively low [59] and spontaneous variations in the BLV genome have a limited impact on the biological properties of the virus [60]. Thus, host factors are thought to play crucial roles in determining the BLV infection profile. Herein, we investigated the role of the host protein PRMT5 in BLV infection in vivo and in vitro. Our study revealed three major conclusions as follows. First, our data suggested that PRMT5 overexpression is involved in the development of BLV infection with a high proviral load, thus affecting which infected cows progress from the asymptomatic stage to the lymphoma stage, and that PRMT5 overexpression may serve as an index of disease progression in vivo. Second, we demonstrated that PRMT5 acts as a novel negative regulator of BLV protein expression in vitro. Knockdown PRMT5 or its inhibition with an inhibitor enhanced BLV gene expression at the RNA and protein levels in BLV-infected cell lines. Finally, CMP5 inhibition demonstrated that the gp51 glycosylation pattern was altered under CMP5 treatment among different BLV-producing cell lines, thereby decreasing BLV-induced syncytium formation mediated by Env glycosylation. Our findings provide insight into the role of PRMT5 in the BLV viral life cycle and for the development of new anti-BLV drugs.

In this study, we found that PRMT5 was significantly upregulated in BLV-infected cattle with HPVL (PVL >10,000 copies/10^5^), but not in those with LPVL (PVL ≤10,000 copies/10^5^) (Figure 1). This finding indicates that PRMT5 upregulation is related to transmission. Furthermore, this upregulation continued to the lymphoma stage (Figure 3). We also performed experimental infection of five cattle carrying the susceptible alleles BoLA-DRB3*1601/*1601, which are associated with an increased risk of developing BLV infection with HPVL, and we confirmed that PRMT5 is upregulated in response to BLV infection (Figure 2A) and its level of upregulation was strongly correlated with BLV PVL (Figure 2B). Finally, we found that PRMT5 upregulation starts as early as two weeks post-infection (Figure 2A). This finding supports previous observations that PRMT5 expression increases at 4–8 days post-EBV infection [46]. Taken together, our data suggest that overexpression of PRMT5 contributes to the development and maintenance of BLV infection. Notably, PRMT5 was not the only PRMT member to be upregulated in BLV infection. PRMT1, which is Type I PRMT, was also upregulated. Additionally, our preliminary data showed that the PRMT1 expression fold-change was correlated with BLV PVL (data not shown). This indicates that PRMT5 is not specifically upregulated, but rather overexpression of other PRMTs is involved in regulating the BLV proviral load.

Our present study clearly demonstrates that PRMT5 negatively regulates BLV expression as PRMT5 inhibition including siRNA-mediated PRMT5 knockdown and treatment with CMP5 enhanced BLV gene expression at the transcriptional and protein levels in BLV-expressing cell lines, indicating that PRMT5 had an additive inhibitory effect on BLV gene expression (Figure 4, Figure 5 and Figure 6). This result is supported by previous data showing that shRNA-mediated reduction in PRMT5 protein levels or its inhibition by a small molecule inhibitor (PRMT5i) in HTLV-1-infected lymphocytes resulted in increased viral gene expression [44]. PRMT5 catalyzes symmetric dimethylation of arginine residues in several histone and non-histone proteins [33,61]. Therefore, PRMT5 may regulate BLV gene expression either via an epigenetic mechanism and by producing repressive histone marks or methylation of non-histone proteins such as transcription factors. Additionally, our findings suggest why BLV maintains a silent state within infected cells in vivo. Indeed, PRMT5 is overexpressed in BLV-infected animals in vivo (Figure 1, Figure 2 and Figure 3). This proviral latency represents a strategy for avoiding the host immune system and consequently allows for tumor development. Thus, numerous studies have focused on identifying compounds capable of reversing BLV latency to enhance immune clearance of the virus; previous studies also revealed histone deacetylation and DNA hypermethylation as epigenetic modifications involved in BLV transcriptional repression [62,63,64]. Therefore, understanding the mechanism of how PRMT5 regulates BLV expression requires further analysis.

Alinari and co-workers [46] investigated the role of PRMT5 in EBV-induced B-cell transformation and developed a small-molecule PRMT5 inhibitor (CMP5) capable of blocking EBV-driven B-cell transformation without affecting normal B cells. Interestingly, we found that CMP5 treatment of FLK-BLV and PK15-BLV affects the glycosylation pattern of BLV gp51. This was further supported by the finding that CMP5 treatment enhanced the formation of complex or hybrid types of *N*-glycan over the high-mannose type. Therefore, following CMP5 treatment, gp51 traveled more slowly than untreated gp51 in SDS-PAGE. The effects of CMP5 on gp51 glycosylation processing involve a sophisticated process requiring a large number of enzymes and organelles, named as the glycosylation machinery. The glycosylation machinery is a group of enzymes, chaperones, transporters, sugar donors, and accessory molecules necessary to form a specific glycan structure [65]. Therefore, glycosylation is a highly regulated and dynamic process that is extremely sensitive to changes in components of the glycosylation machinery. The diversity of glycans depends on not only the expression levels of the molecules involved in glycan biosynthesis, including glycosyltransferases, but also on the interplay of all regulatory molecules involved in the process. This may affect both the number of glycans (macroheterogeneity) and nature of these glycan chains (microheterogeneity) [65]. Thus, CMP5 treatment may alter the expression levels of some components of the glycosylation machinery, thereby altering the gp51 glycosylation pattern.

Notably, the effect of CMP5 treatment on the gp51 glycosylation pattern differs between the two cell lines (FLK-BLV and PK15-BLV), which belong to different host species. In the PK15-BLV cell line, CMP5 treatment enhanced the formation of the complex type of *N*-glycan over the high mannose type. In FLK-BLV, however, the results did not reveal that the irregular type of *N*-glycan resulted from CMP5 treatment. Similarly, a previous report clearly showed distinct differences in the glycosylation profiles of HIV-1 ENV gp120 expressed in CHO, which originated from hamsters, and 293T cells, which originated from humans [66]. Therefore, this variation in different cell lines is expected as the nature of *N*-linked glycans attached to a glycoprotein is determined not only by the peptide backbone of a protein, but also by the cell in which it is expressed.

Although CMP5 affects the gp51 apparent molecular weight, siRNA knockdown of PRMT5 did not appear to induce the same effect (Figure S1). The reason why this difference arose between siRNA-mediated PRMT5 knockdown and CMP5 treatment was unclear, but there are two possibilities. First, the impact of CMP5 treatment on gp51 glycosylation may be non-specific, resulting from inhibition of other PRMTs protein rather than PRMT5. Second, gp51 glycosylation is a highly dynamic process that is highly influenced by the time and efficiency of PRMT5 inhibition and differs between pharmacological inhibition and knockdown inhibition. Considering that Alinari and co-workers [46] showed that CMP5 selectively blocks S2Me-H4R3 in JeKo cells, whereas it is inactive against PRMT1, PRMT4, and PRMT7 in cellular screening assays, CMP5 appears to be selective for PRMT5 over type1 PRMTs. Additionally, the initial predicted binding interactions of CMP5 with their hPRMT5 model suggested that the pyridine ring of CMP5 forms a p-stacking interaction with the Phe327 residue, which is critical for directing PRMT5 to catalyze symmetric dimethylation of arginine, explaining the selectivity of CMP5 for type II PRMT5, not type I PRMTs. They also performed RNA-seq of PRMT5-shRNA or CMP5 treatment in transformed LCLs (60A) cells and observed significant overlap between genes de-repressed by CMP5 and genes de-repressed by PRMT5-shRNA, confirming the specificity of this compound. In contrast, we observed that CMP5 treatment increases the apparent molecular weight of gp51 at 20 µM, but not at 5 or 10 µM (Figure 6b). Therefore, the extent and duration of PRMT5 inhibition in siRNA knockdown may be insufficient to affect the glycosylation processing of gp51. Further transcriptomic or proteomic studies are required to identify the differentially expressed genes and determine the biological processes altered by CMP5 treatment or PRMT5 knockdown.

Our study showed that CMP5 treatment impeded BLV-induced syncytium formation, which was measured by LuSIA [8,51]. However, CMP5 treatment did not impair the gp51 expression at the cell membrane (Figure 8C,D). Seemingly, the intracellular expression of Env accumulated near to the nucleus after CMP5 treatment (Figure 8B). The reason of this phenomena and whether or not it is related to the observed reduction of the syncytia formation remain unclear. On the other hand, previous studies reported that the electrophoretic mobility of the surface subunit of the HIV envelope (gp120) was decreased when gp120 was synthesized in the presence of castanospermine or 1-deoxynojirimycin (inhibitors of glucosidase I). Consequently, these inhibitors blocked HIV-1-induced syncytium formation and cytopathicity [58,67,68]. Therefore, incorrect glycosylation processing of gp120 may negatively affect HIV-1 induced syncytium formation. In addition, BLV SU-linked *N*-glycosylation appeared to regulate the syncytium-forming capacity in vitro [26,27]. Taken together, CMP5 treatment likely impedes BLV-induced syncytium formation by affecting gp51 glycosylation processing.

In conclusion, we determined various roles for PRMT5 in BLV infection in vivo and in vitro; CMP5 and other PRMT5 inhibitors should be further investigated and evaluated in clinical studies as a novel antiretroviral therapy.

## Supplementary Materials

The following are available online at https://www.mdpi.com/1999-4915/12/6/650/s1, Table S1: Primer sequences for qRT-PCR used in the study, Figure S1: Effect of PRMT5 knockdown on gp51 electrophoretic mobility. Figure S2: Effect of PRMT5 knockdown and CMP5 treatment on Gag processing.
