# Supplementary material for: PRMT5 Is Required for Bovine Leukemia Virus Infection In Vivo and Regulates BLV Gene Expression, Syncytium Formation, and Glycosylation In Vitro
Source: Viruses. 2020 Jun 16;12(6):650. doi: 10.3390/v12060650 (PMC7354529; doi:10.3390/v12060650)
Supplement: Supplementary file 1 [file viruses-12-00650-s001.pdf]

viruses

MDPI

# Supplementary Materials: PRMT5 Is Required for Bovine Leukemia Virus Infection In Vivo and Regulates BLV Gene Expression, Syncytium Formation, and Glycosylation In Vitro

**Table S1.** Primer sequences for qRT-PCR used in the study.

| Gene | Forward 5′→3′ ⚜ | Reverse 5′→3′ | Accession Number |
|---|---|---|---|
| PRMT5 cattle | CCTGAATTGCGTCCCCGAAA | TGCAGAGGAAATCAAACCCCT | NM_001105374.1 |
| PRMT5 FLK-BLV | TCCTCCATGTTCTGGATGCG | GTGTGAGAAGTTGGTGCGT | XM_012181004.2 |
| PRMT5 PK15-BLV | CCTGAATTGCGTCCCCGAAA | TGCAGAGGAAATCAAACCCCT | NM_001160093.1 |
| *Gag* (p24) | GACCAAACGGCCCATATGAC | TTGGGCTGAGCTGATTGTTG | DDBL: EF600696 [BLV] [Ref 67] |
| *Tax* | TGGAACAACTTAGTAACGCATC | GCTCGCCTAGGGGTAGAATAC | DDBL: EF600696 [BLV] [Ref 67] |
| GAPDH cattle | TTCAACGGCACAGTCAAGG | ACATACTCAGCACCAGCATCAC | NM_001034034.2 |
| GAPDH FLK-BLV | TGGTGAAGGTCGGAGTGAAC | ACGATGTCCACTTTGCCAGT | NM_001190390.1 |
| GAPDH PK15-BLV | CTGAGACACGATGGTGAAGG | ACAATGTCCACTTTGCCAGA | NM_001206359.1 |

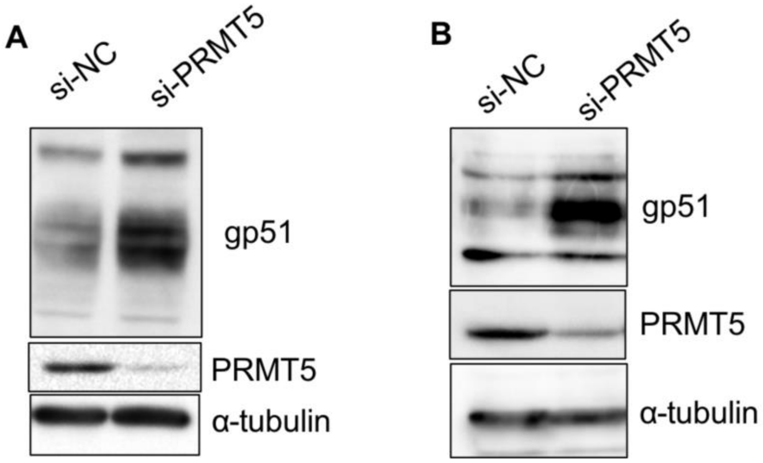


**Figure S1.** Effect of PRMT5 knockdown on gp51 electrophoretic mobility. (**A**) PRMT5 knockdown of FLK-BLV cell line using scramble siRNA (si-NC) or siRNA targeting PRMT5 (si-PRMT5) at 4 nM for 48 h. (**B**) PRMT5 knockdown of PK15-BLV cell line using scramble siRNA (si-NC) or siRNA targeting PRMT5 (si-PRMT5) at 2 nM for 72 h.

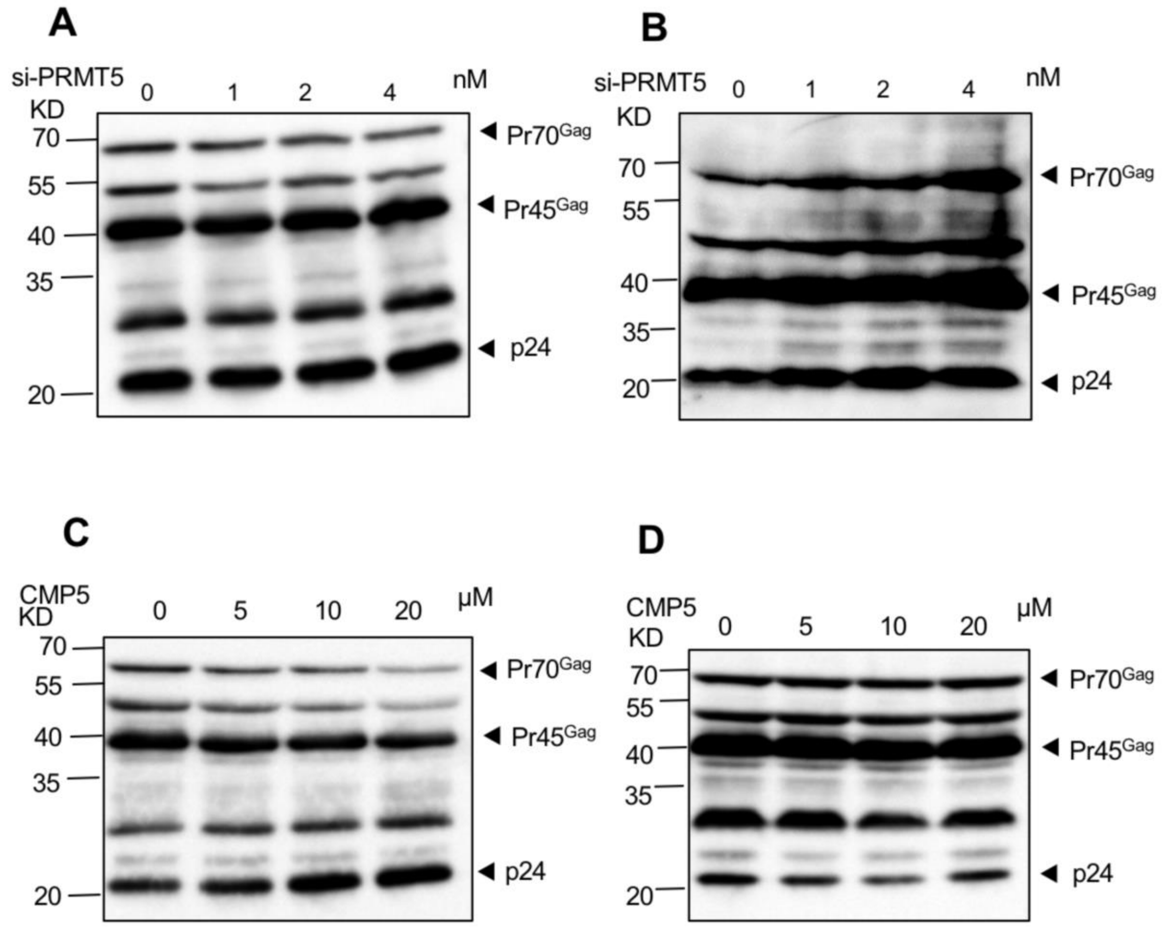


**Figure S2.** Effect of PRMT5 knockdown and CMP5 treatment on Gag processing. (**A**) Complete western blot image of Gag protein of data shown in Figure 5A. (**B**) Complete western blot image of Gag protein of data shown in Figure 5B. (**C**). Complete western blot image of Gag protein of data shown in Figure 6A. (**D**) Complete western blot image of Gag protein of data shown in Figure 6B. Positions of BLV p24, $Pr45^{Gag}$ and $Pr70^{Gag}$ are indicated.
